# A New Approach to Quantifying Muscular Fatigue Using Wearable EMG Sensors during Surgery: An Ergonomic Case Study

**DOI:** 10.3390/s23031686

**Published:** 2023-02-03

**Authors:** Johan Merbah, Bertrand R. Caré, Philippe Gorce, François Gadea, François Prince

**Affiliations:** 1 International Institute of Biomechanics and Occupational Ergonomics, 83400 Hyères, France; 2 BERGIA Solutions, 83000 Toulon, France; 3 International Institute of Biomechanics and Occupational Ergonomics, Université de Toulon, STAPS, CS60584, 83041 Toulon, France; 4 Département de Chirurgie, Faculté de Médecine, Université de Montréal, Montréal, QC H3C 3J7, Canada

**Keywords:** musculoskeletal disorders, wearable sensors, fatigue, biomechanics, surgery, orthopedics, ergonomics, electromyography (EMG), position

## Abstract

(1) Background: Surgeons are exposed to musculoskeletal loads that are comparable to those of industrial workers. These stresses are harmful for the joints and muscles and can lead to musculoskeletal disorders (MSD) and working incapacity for surgeons. In this paper, we propose a novel ergonomic and visualization approach to assess muscular fatigue during surgical procedures. (2) Methods: The activity of eight muscles from the shoulder girdle and the cervical/lumbar spines were evaluated using position and electromyographic wearable sensors while a surgeon performed an arthroscopic rotator-cuff surgery on a patient. The time and frequency-domain variables of the root-mean-square amplitude and mean power frequency, respectively, were calculated from an electromyographic signal. (3) Results: The entire surgical procedure lasted 73 min and was divided into 10 sub-phases associated with specific level of muscular activity and fatigue. Most of the muscles showed activity above 60%, while the middle trapezius muscles were almost constantly activated (>20%) throughout the surgical procedure. (4) Conclusion: Wearable sensors can be used during surgical procedure to assess fatigue. Periods of low-to-high activity and fatigue can be evaluated and visualized during surgery. Micro-breaks throughout surgical procedures are suggested to avoid fatigue and to prevent the risk of developing MSD.

## 1. Introduction

For several years, workplace have seen the emergence of the “well-being at work” trend [1]. The hospital sector is no exception to this. The surgical community has conducted research to identify parameters related to the risk of developing musculoskeletal disorders (MSDs). Equipment, as well as technical and operating conditions, have improved for both open and minimally invasive surgical procedures [2,3,4] to prevent the risk of MSDs [5,6]. These MSDs affect muscles, tendons, nerves, and ligaments and are associated with symptoms ranging from localized fatigue and slight discomfort to significant pain [7]. In France alone, MSDs account for 87% of occupational diseases, with an associated cost of more than EUR 2 billion per year [8]. A systematic review and meta-analysis [9] reported many MSDs among surgical practitioners and interventionists, resulting in practice restrictions and career interruptions creating a potential workforce shortage by 2025 [10]. Self-reported symptoms based on a questionnaire administered to 39 residents reported pain in the neck (59%), lower back (55%), upper back (35%), and shoulders (34%) [11]. The number of surgeons impaired by work-related pathologies directly attributable to their surgical activities is increasing [9]. This places surgeons at greater risk of developing MSD compared to other professions in both the public and private sectors. The causes of MSD in surgeons are mainly related to physical loads and time constraints [2,12]. Ergonomists have compared surgeons with industrial workers [13] and reported that surgeons experience much more severe environmental- and working-stress conditions than industrial workers.

The repetitive aspect of tasks causing fatigue, the poor ergonomic design of tools, the prolonged immobility and awkward postures, the loads moved, as well as the extreme concentration required by the high precision of gestures are all proven causes associated with MSDs in workers [13,14,15,16]. This fact is not new; however, few research works have tried to characterize surgical tasks from an ergonomic point of view. Kant et al. [4] showed that surgeons and operating assistants adopted risky postures due to the static and long-lasting nature of their work. Higher ergonomic-stress loads were reported in general surgeons compared to ear-nose-and-throat (ENT) specialists [4]. In addition, general surgeons more often place their backs in bent, twisted, or combined positions compared to ENT surgeons, who generally used a sitting position for surgery. In addition, many assessments of medical practice are based on questionnaires rather than on quantitative, objective biomechanical data [3,5,9,17].

It would therefore seem that many health practitioners, especially surgeons, regardless of their specialty, suffer from MSDs, which affect their capacity for intervention [2]. Furthermore, recently introduced minimally invasive procedures in surgery have changed practices, particularly in terms of postures and ranges of motion [18], but continue to cause MSDs as they violate several ergonomic principles [19].

It appears that in minimally invasive surgery, movements require more precision and concentration compared to open surgery, but the latter is more physically demanding [20]. The variations in movement are reduced and the posture is maintained mostly static with isometric muscular contraction, and for a longer period of time, which increases the physical constraints on the body [17,21,22,23].

Recently, surface electromyography (EMG) was used to quantify physical stress during minimally invasive neurosurgery and showed that an optimal surgical-device configuration was able to decrease carpi radialis muscular fatigue [24]. In a study using the Rapid Upper Limb Assessment (RULA) [24] and surface EMG, researchers found a significant difference in both RULA and EMG results between optimal and non-optimal body positioning while performing simulated laryngeal surgical procedures [25]. Finally, another study used the mean power frequency (MPF) of EMG signals as a fatigue variable, and it showed that decreases in both the MPF and the root mean square (RMS) amplitude of EMG signals were associated with muscular fatigue in static and non-demanding positions adopted by laparoscopic surgeons [25]. Unfortunately, the articles presenting EMG activity and muscular fatigue never show the EMG time series and only the means and standard deviations are provided.

In this context, the purpose of this study is to quantify and visualize both muscular activity and muscular fatigue using wearable sensors throughout a complete surgical procedure.

## 2. Materials and Methods

The surgeon and the patient were informed of the purpose, methodology, and potential risks of the study before giving verbal and written informed consent. No additional risk was identified for the patient. The study was conducted in accordance with the Declaration of Helsinki [26], and approved by the Ethics Committee of St-Roch Hospital, Toulon, France (protocol #S-ERG-2021-001, approved on 4 January 2021) for studies involving humans. The inclusion criteria for the surgeon were to have no known pathology and absence of neurological or orthopedic pain. The surgeon was a right-handed, 37-year-old male. He was 181 cm tall, and his mass was 75 kg. The surgery performed was a right-shoulder-rotator-cuff reinsertion under arthroscopy on a 60-year-old female patient diagnosed with a rotator-cuff tear resulting from a fall. The patient was an active person working as a geriatric nurse. The rotator-cuff surgical procedure was carried out with the double-row-repair approach [27,28,29]. The EMG signals were recorded bilaterally from muscles most solicited during the surgery, namely the medial deltoid (shoulder), the upper and middle trapezius (cervical spine) and latissimus dorsi (lumbar spine) muscles. The surgeon announced the chronological sequences of the different phases of the surgical procedure.

### 2.1. Material

Table 1 shows the patient set up, as well as the surgical tools and control devices required for this specific surgery.

### 2.2. Data Acquisition and Analysis

**Figure 1:**
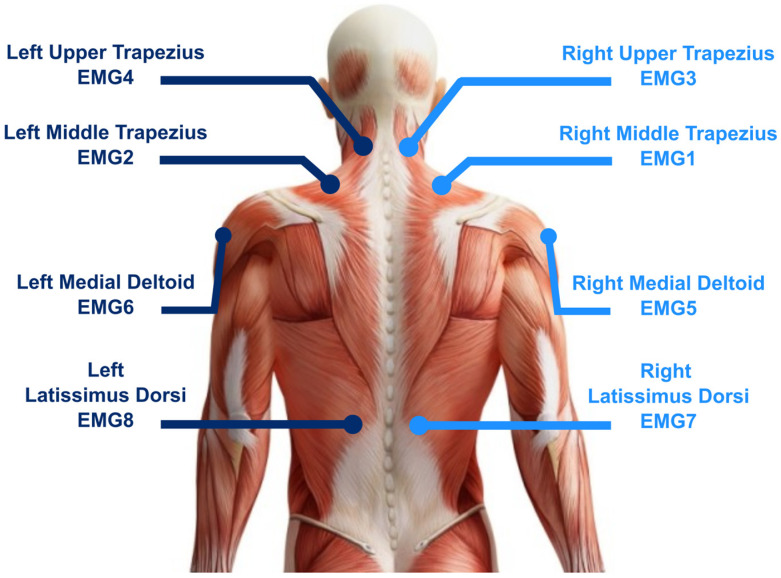
Positioning of the EMG sensors on specific muscles.

This study consisted of monitoring the EMG activity of 8 different muscles while performing a rotator-cuff surgery under arthroscopy. Specific positions of EMG sensors are illustrated in Figure 1. Surface EMG was recorded using Trigno™ Avanti Sensors (DELSYS Inc., Natick, MA, USA) sampled at 1778 Hz. Before securing the sensors with a double-sided hypoallergenic adhesive, the skin was shaved, slightly abraded, and cleaned with alcohol. The EMG was captured on muscle belly based on protocol described by Delsys [30].

**Figure 2:**
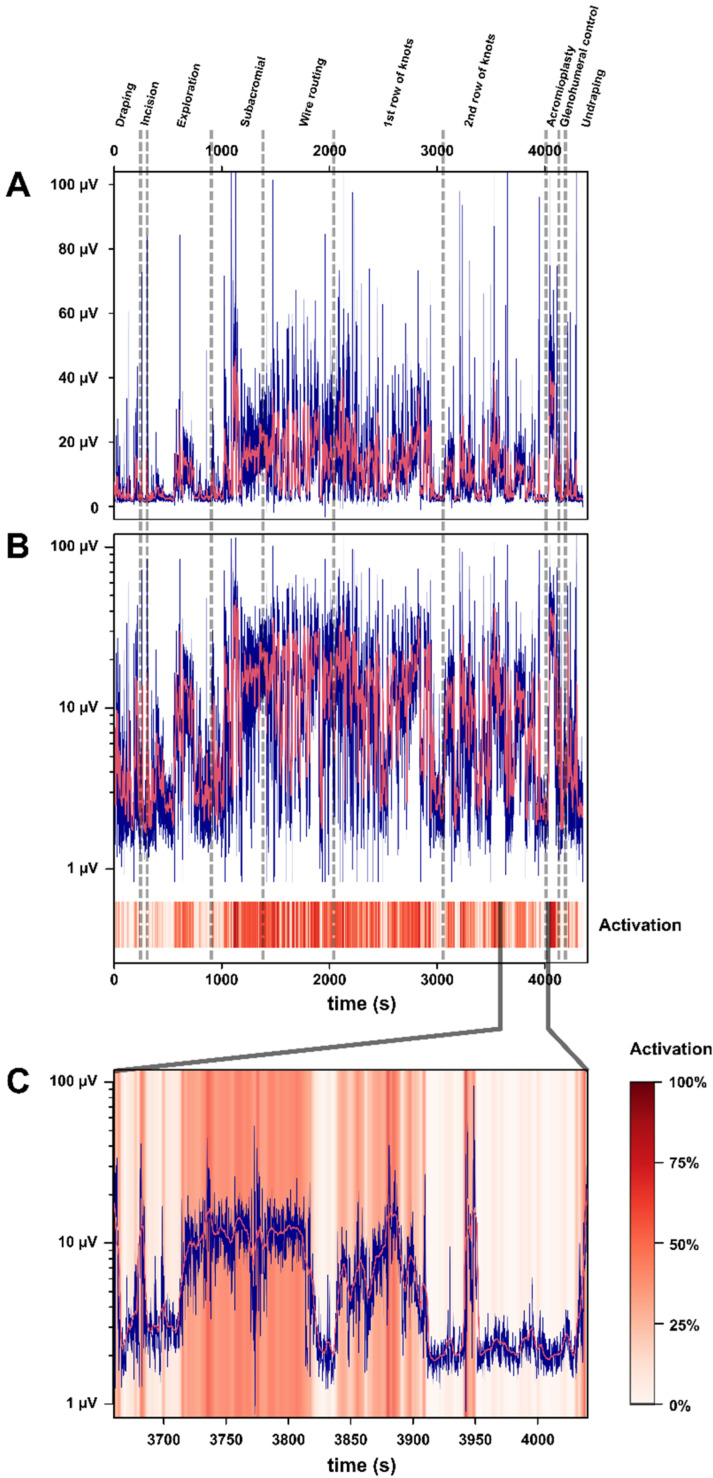
Illustration of the passage from raw EMG signal to intelligible activation signal. (**A**). Filtered EMG signal of the right upper trapezius (blue line) in µV during the whole surgical-procedure recording (0 to 4380 s, *x*-axis). The moving-average-smoothed signal is also shown (red line, window: 5 s). The different surgery sequences are marked as vertical dashed lines. (**B**) The same filtered EMG signal from the right upper trapezius sensor is shown in logarithmical scale (from 1 µV to 100 µV) for the whole duration of the surgical-procedure recording. Corresponding activation signal color-coded from white (0%) to dark red (100%) is shown below the EMG signal. (**C**) Zoom between 3650 s and 4050 s (end of the second row of knots) of the right upper trapezius signal (in logarithmic scale as in B) that illustrates the on/off nature of the muscular effort: muscle activation oscillates between low-intensity plateaus around 1–2 µV, moderate-to-high-intensity plateaus around 10 µV, and high-intensity peaks above 30 µV. Background is color-coded according to the activation percentage, with the scale on the right side of C matching the numerical scale on the left side of the graph (1 µV → 0%, 10 µV → 50%, 100 µV → 100%).

The raw EMG signal was initially high-pass filtered (20 Hz tenth-order Butterworth). Next, a rectification and a low-pass filter (500 Hz third-order Butterworth) were applied. For visualization purposes, a sliding average (0.5-second window) was applied to obtain the signal envelop. For RMS and MPF computations, the filtered EMG signals were used (without the moving average) for each muscle and for the different surgical phases. Filtered EMG signals are difficult to interpret in terms of effort intensity of muscle activation (Figure 2A). While inspecting the filtered EMG data, we found that informational content of the signals was between 1 µV and 20 µV but was visually squashed because of signal peaks around 100 µV. We also observed that all filtered EMG signal values were between 1 µV and 100 µV. Consequently, to better visualize EMG signal variations in the range of 1–20 µV while conserving the over-50 µV -signal-peak information, we used a logarithmic scale (Figure 2B) that revealed otherwise-elusive muscle-activation patterns in the 1–20 µV range. We therefore computed a visual representation that preserves the most important muscle information and is easier to understand for medical professionals who are unfamiliar with it. To take advantage of the capacity of the human eye to detect subtle changes, we also proposed a color-coded scale (Figure 2C) with a signal value of log10 (1 µV) corresponding to white (0%, basal activation), and log10 (100 µV) to red (100% maximal observed muscle activation). This yielded a visual representation of the muscle activation that combines multiple advantages: (1) it is normalized, and it can be compared between the different muscle groups; (2) it is easy to interpret functionally as color-coded low-intensity/mid-intensity/high-intensity phases; and (3) it retains the information content for all the signal-range values across two orders of magnitude.

## 3. Results

### 3.1. Surgical Phases and Time

The surgical procedure, including the undraping, lasted 4380 s (1h13). The duration of each stage is expressed as a percentage of the total time of the data recording (Table 2). The entire surgical procedure was divided into 10 distinct stages. These stages were signaled by the surgeon’s announcement during the surgery. The time was noted and allowed us to make this temporal partition. The exploration, subacromial, and wire routing represented 14%, 11%, and 15%, respectively, of the total duration. The execution of the first and second row of nodes were the longest phases, representing 23% and 22% of the surgical procedure, respectively. It is noteworthy that the draping and the undraping phases together represented 10% of the total duration of the surgical procedure (Table 2).

### 3.2. EMG Amplitude and Fatigue

**Figure 3:**
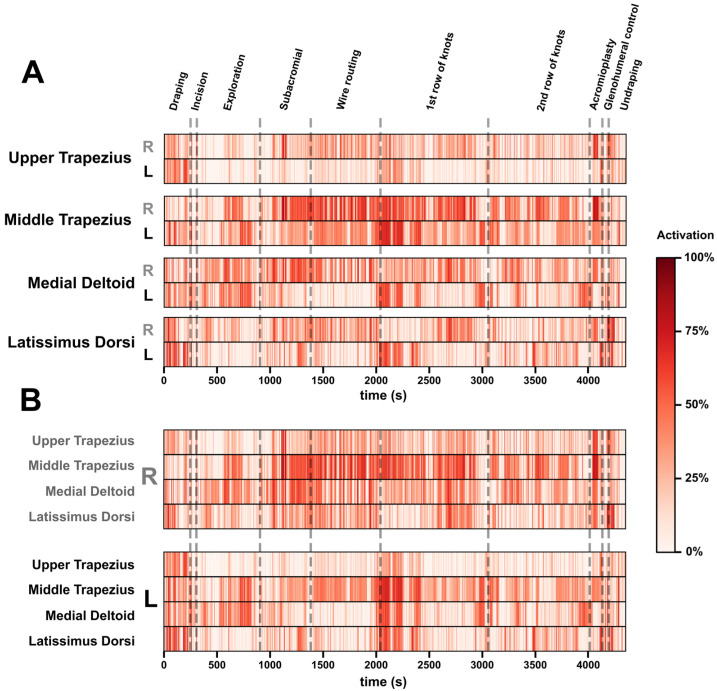
Color-coded muscle activation recorded during the surgical procedure. Time increases from left to right. (**A**) Activation signals grouped by muscle (right and left adjacent for each muscle group) from the anterior to the posterior, for easier functional interpretation. (**B**) Activation signals but grouped by laterality (all right muscles in grey, all left muscles in black) for easier asymmetry detection. Colours range from white (0%) to dark red (100%). The different surgery stages are marked as vertical dashed lines.

Muscular activity from eight muscles was processed as described above and the logarithmic representation (Figure 3) was used to visualize the fluctuation during the 10 phases of the surgery. The lighter the colours, the less activated the muscle is in relation to its maximum activation during the surgery. The results showed that the bilateral middle trapezius muscles were constantly activated above 20% throughout the surgical procedure. The wire routing and the first row of knots were quite demanding, with an activation level above 50% of the maximum during the surgery. The most significant activation of the entire surgical procedure was for the right-middle-trapezius muscle. This occurred briefly in the middle of the “subacromial” phase and for a longer period during most of the acromioplasty phase, during which the muscle activation was above the 65% threshold. The bilateral upper-trapezius muscle activation seemed constant (0–20%) across the surgical phases, except for the draping and end of the surgery, where it reached above 40% of the maximal activation. In addition, the left side at the draping stage and the right side during the glenohumeral control stage reached levels of activation above 60%. During the first row of knots, the left-middle-trapezius muscle was the only one to reach a persistent and high activation level above 60%. The left and right latissimus dorsi muscles were activated in a moderate-to-sustained manner throughout the surgery, with some periods in which the activation was greater than 50% of the maximum. The left side seemed to have more peaks of activation above 50%, but also more periods of activation below 25%. The right side, on the other hand, seemed to be constantly activated above 25%.

To a lesser extent, the same was true for the other muscles. Finally, most muscles, including the right upper and middle trapezius, left medial deltoid muscles and bilateral latissimus dorsi showed some muscle activity above 60%. Figure 3B shows that all the muscles were also more active on the right side than on the left side.

Concerning the analysis of the EMG signal by means of the RMS, which allows each muscle or for each phase to visualize and quantify the amount of muscle activity, the right medial deltoid seems to have been the most active muscle, with an average of 27.3µV over the whole procedure (Table 3). In terms of RMS, for the phases described, draping, acromioplasty, and glenohumeral control seem to have been very demanding stages for the surgeon’s muscles, with an average activation per phase of 13 µV, 8.7 µV, and 10.8 µV respectively. During acromioplasty, the right middle trapezius, and the right medial deltoid muscles present high RMS values, of 31.3µV and 28.3µV, respectively. The same was true for the subacromial phase, during which the right medial deltoid muscle reached 36.8 µV. Only the exploration stage was clearly less demanding from a muscular-activation point of view. Regarding the draping stage, with the higher RMS values, the average reached 13.0 µV. During this part of the surgery, only the left and right upper trapezius presented low levels of muscular activation (<6 µV). The right and left medial deltoid were highly active, with values of 32.3 µV and 24.7 µV, respectively.

We then examined the relative decreases in MPF (in %, Table 4) as a marker of muscle fatigue for each of the eight muscles recorded. The literature reports that a drop of 8% or greater in MPF after a task is associated with significant neuromuscular fatigue [31]. We therefore computed, for each task and each muscle, the MPF using the filtered signal of the first 3 s of the stage, and the MPF using the filtered signal of the last 3 s of the stage. In addition, we computed the relative difference as a percentage. The drops in MPF greater than 8% during any stage are highlighted in red. These drops were seen primarily in the middle of the procedure. The draping, exploration, glenohumeral-control, and undraping phases caused relatively little muscle fatigue. The surgeon experienced the greatest fatigue during five steps in the middle of the procedure, namely the subacromial, wire-routing, first-row-knots, second-row-knots, and acromioplasty phases. The fatigue level of the right-middle-trapezius muscle reached −47.2% during the subacromial stage. In general, the upper trapezius muscles were the most fatigued, with values <−8%. The fluctuation of the percentage towards positive values throughout the surgical procedure indicates periods of rest or movements with large ranges of motion.

## 4. Discussion

The objective of this study was to quantify and to visualize muscular activity and to quantify muscular fatigue using wearable sensors during a surgical procedure. Wearable sensors are well tolerated and give valuable information during surgical procedures [32,33]. The EMG recordings of the eight muscles were divided into 10 different surgical phases, allowing an analysis of the most heavily solicited muscles using RMS and MPF parameters. The visualization of EMG time series using a log scale yields an easier characterization of the signal compared to traditional EMG visualizations, as it allows both lower and higher amplitudes to be visualized at the same time [34,35,36]. The bilateral middle trapezius muscles were the most heavily activated muscles during the surgery, and they were constantly activated above a 20% threshold. This was probably due to the constant attention paid by the surgeon to his upper limbs to avoid touching the sterile operative field, as well as to the constant manipulation of the surgical tools and the patient’s arm. This high muscle-activation level without any rest periods is clearly associated with increased physical stress and joint load. The phases following incision are mostly static phases associated with isometric contractions. Even if these stages are interspersed with some actions (picking up tools, moving the table backwards or repositioning the patient’s upper limb), most of them can be considered almost static. It is clear, from an ergonomic point of view, that these phases are potentially associated with MSDs of the upper and lower back and the neck, as seen in a previous report [37]. In both minimally invasive and robotic surgery, a decrease in MPF seems to indicate an increased muscular fatigue, and lower contraction amplitudes [31,36]. The maximum muscular activity of each muscle during surgery was chosen to perform the normalization expressed as a percentage of this maximum and was used in other studies [38,39,40]. In terms of muscle activity, RMSs provide relevant information about muscle activation. The draping and exploration phases had relatively high RMS values. This can be explained by the dynamic and wide movements performed by the surgeon. In addition, the medial deltoid (left and right) and the middle trapezius (left and right) were the four most active muscles during the whole surgery from the point of view of RMS.

Muscle fatigue was shown to be minimal during the draping and undraping. These phases can be considered as mobile, in contrast to static phases with isometric contractions. These periods were chosen as references for comparing the other and more precise surgical phases. Persistent fatigue was seen for the right upper limb with significant residual fatigue shown by the upper and middle trapezius, medial deltoid, and latissimus dorsi muscles. This could explain the muscular fatigue accumulated during the surgical procedure, in which all the muscles were solicited with more than 30% of their maximum capacity for more than half of the surgical duration. Prolonged muscular load beyond 10% of the maximum voluntary capacity results in less oxygen perfusion in the muscle and may lead to discomfort resulting from ischemia [41,42]. Intraoperative breaks or micro-breaks have been shown to be beneficial on surgeon physical function, fatigue, stress, and concentration [43,44,45,46], as in other workers. It seems that the level of concentration required according to the stage of surgery is an important notion from a muscular-activity point of view. As the risk increases, it seems that the surgeon contracts his muscles more, in isometric mode, because the precision of their gestures must be perfect. These phases are critical from an ergonomic point of view. It should be noted that ample movements elicit large muscle-activation signals, but are not necessarily the actions that induce the most neuromuscular fatigue. By contrast, isometric quasi-static muscular contractions for prolonged periods of time do not appear as large muscle-activation signals, but they are the activities that induce the greatest MPF drops, and, hence, the most neuromuscular fatigue.

As this was a case study, this work investigated the performance of a single surgeon performing a standard arthroscopic procedure without complications. The results highlighted here suggest, as underlined by the existing literature, the deleterious effects of mini-invasive surgical procedures. In this context, our ergonomic analysis of this technical procedure highlighted gestures presenting a high risk of MSD. However, it concerned only one patient and only one surgeon, using one technique and one type of material. It should therefore be noted that similar studies should be undertaken and reproduced before generalizing these findings. No maximum voluntary contraction was performed. The maximum of each muscle during surgery was chosen to perform the normalization of the signal intensity. The goal of future research is to increase the number of surgeons, the number of patients, and the type of surgery, with an initial focus on minimally invasive surgeries and orthopedic procedures. The time for each phase of the operation (Table 2) could vary, depending on the surgeon and the operation itself. This methods of expressing surgery by phases was chosen arbitrarily in order to be able to discuss the results according to the tasks performed.

We hope to pave the way for more comprehensive and statistically valid research that could be applied to all types of jobs. In addition, the EMGs used were combined with IMUs. The logical next step would be to conduct a combined kinematic and EMG data analysis, such as by acquiring joint-articulation-angle data synchronized with muscular EMG signals in order to yield more powerful results from an ergonomic perspective.

## 5. Conclusions

Based on these results, a surgeon performing a rotator-cuff procedure experienced muscular fatigue levels that varied throughout the different phases of the surgery. This study provided a new visual representation of muscular activity with associated color-coded descriptions of low/mid- and high-intensity-muscular-activity phases. The RMS and MPF values also provided the phases in which the surgeon experienced the most fatigue throughout the surgical procedure. With this information, we recommend including three to four short breaks (<60 s) during surgery to avoid fatigue accumulation. The increased mobility of these muscular regions would allow revascularisation, remove physical stress, and reduce the risk of developing MSDs.

## Figures and Tables

**Table 1:** Surgical Set-up.

Patient Setup	Surgical Instruments	Control Devices
Schaerer 400 Operating Table	Smith & Nephew Rotator-cuff box	Sheaver Stryker 4 mm/ 30° Optics + column with Stryker screen
Patient’s upper limb placed on a Spider Smith & Nephew articulated arm	Spikes	
	Anchors Detangling forceps Anchor footprints Wire cutters Gripping pliers Crocodile clip Hammer Pine pike Sheaver Stryker 4 mm/30° optics + column with Stryker screen Stryker formula core Sheaver orthopedic box for opening/closing and trocar for optics	

**Table 2:** Description of surgical stages, their corresponding time and % of surgical time.

Start (s)	Stop (s)	Stage of Surgery	Duration (% Total)
0	240	Draping	6
240	300	Incision	1
300	900	Exploration	14
900	1380	Subacromial	11
1380	2040	Wire Routing	15
2040	3060	1st row of knots	23
3060	4020	2nd row of knots	22
4020	4140	Acromioplasty	3
4140	4200	Glenohumeral Control	1
4200	4380	Undraping	4

**Table 3:** RMS values (µV) from eight muscles during the different stages of the surgery.

	Draping	Incision	Exploration	Sub-Acromial	Wire Routing	First-Row Knots	Second-Row Knots	Acromio-Plasty	Gleno Humeral Control	Un-Draping
R. Middle Trapezius	8.6	8.9	10.2	23.0	24.3	21.0	15.1	31.3	9.6	12.0
L. Middle Trapezius	20.5	13.2	12.9	9.2	15.8	21.0	12.1	13.4	12.9	15.5
R. Upper Trapezius	5.5	3.7	3.5	4.9	5.6	4.9	4.3	7.0	5.9	4.9
L. Upper Trapezius	5.8	3.4	2.9	3.0	3.4	3.6	3.1	4.2	3.8	4.2
R. Medial Deltoid	32.3	27.5	27.5	36.8	28.3	22.6	20.3	28.3	25.0	24.6
L. Medial Deltoid	24.7	22.0	20.0	10.8	10.3	18.7	16.1	13.1	19.7	24.9
R. Latissimus Dorsi	9.0	4.6	5.2	7.4	8.0	6.6	5.5	8.5	12.0	9.6
L. Latissimus Dorsi	8.6	8.9	10.2	23.0	24.3	21.0	15.1	31.3	9.6	12.0

**Table 4:** Relative difference in MPF between the beginning and the end of each stage for all muscles expressed in %. The MPF evolutions between the first 3 seconds and the last 3 s of each stage with respect to MPF from the 3 first seconds of each stage are shown. All negative values are highlighted. The values between 0 and −8% are in pink. The values < −8% are in red. A drop of 8% or greater in MPF after a task is considered a robust marker of fatigue in the literature [31].

	Draping	Incision	Exploration	Sub-Acromial	Wire Routing	First-Row Knots	Second-Row Knots	Acromio-Plasty	Gleno Humeral Control	Un-Draping
R. Middle Trapezius	16.8%	4.2%	64.9%	−47.2%	−0.7%	68.7%	32.8%	−24.9%	46.1%	21.4%
L. Middle Trapezius	12.9%	11.1%	−0.9%	−14.1%	−37.5%	−6.3%	80.3%	45.6%	1.8%	−17.0%
R. Upper Trapezius	55.3%	−23.4%	33.6%	−24.8%	26.6%	−26.2%	12.5%	−18.9%	3.7%	17.1%
L. Upper Trapezius	19.8%	−3.6%	25.1%	−10.0%	−9.3%	−3.8%	26.0%	−17.1%	2.6%	12.6%
R. Medial Deltoid	29.5%	18.9%	44.8%	−22.6%	17.5%	43.9%	−34.6%	9.4%	5.1%	90.6%
L. Medial Deltoid	32.1%	29.0%	7.7%	26.5%	−31.1%	−13.6%	22.0%	33.1%	−3.8%	36.0%
R. Latissimus Dorsi	−13.6%	−11.1%	5.5%	2.8%	−3.2%	−28.9%	−1.2%	−14.1%	−6.7%	21.6%
L. Latissimus Dorsi	1.8%	−11.0%	25.4%	−1.6%	9.2%	−16.7%	−18.2%	23.6%	−3.1%	3.4%

## Data Availability

Data available on request due to restrictions e.g., privacy or ethical. The data presented in this study are available on request from the corresponding author.

## References

[1] Ajith S., Sivapragasam C., Arumugaprabu V. (2019). Quantification of risk and assessment of key safety factors for safe workplace in Indian building construction sites. Asian J. Civ. Eng..

[2] Hamilton B.C.S., Nguyen T.C. (2021). We Asked the Experts: Surgical Ergonomics: Stop Suffering in Silence. World J. Surg..

[3] Sweeney K., Mackey M., Spurway J., Clarke J., Ginn K. (2021). The effectiveness of ergonomics interventions in reducing upper limb work-related musculoskeletal pain and dysfunction in sonographers, surgeons and dentists: A systematic review. Ergonomics.

[4] Kant I.J., de Jong L.C.G.M., van Rijssen-Moll M., Borm P.J.A. (1992). A survey of static and dynamic work postures of operating room staff. Int. Arch. Occup. Environ. Health.

[5] Epstein S., Tran B.N., Capone A.C., Ruan Q.Z., Lee B.T., Singhal D. (2018). Work-Related Musculoskeletal Disorders among Plastic Surgeons: A Systematic Review. J. Reconstr. Microsurg..

[6] Bolduc-Bégin J., Prince F., Christopoulos A., Ayad T. (2018). Work-related musculoskeletal symptoms amongst Otolaryngologists and Head and Neck surgeons in Canada. Eur. Arch. Oto-Rhino-Laryngol..

[7] INRS Troubles Musculosquelettiques (TMS). http://www.inrs.fr/risques/tms-troubles-musculosquelettiques/statistiques.html.

[8] Assurance_Maladie Les TMS: Définition et Impact. https://www.ameli.fr/var/entreprise/sante-travail/risques/troubles-musculosquelettiques-tms/tms-definition-impact.

[9] Epstein S., Sparer E.H., Tran B.N., Ruan Q.Z., Dennerlein J.T., Singhal D., Lee B.T. (2018). Prevalence of Work-Related Musculoskeletal Disorders Among Surgeons and Interventionalists: A Systematic Review and Meta-analysis. JAMA Surg..

[10] Tim Dall R.R., Chakrabarti R., Jones K., Iacobucci W. (2020). The Complexities of Physician Supply and Demand: Projections From 2018 to 2033.

[11] Knudsen M.L., Ludewig P.M., Braman J.P. (2014). Musculoskeletal pain in resident orthopaedic surgeons: Results of a novel survey. Iowa Orthop. J..

[12] DREES (2017). Arrêts Maladie Dans le Secteur Hospitalier: Les Conditions de Travail Expliquent les Écarts Entre Professions.

[13] Seagull F.J. (2012). Disparities between industrial and surgical ergonomics. Work.

[14] Anwer S., Li H., Antwi-Afari M.F., Wong A.Y.L. (2021). Associations between physical or psychosocial risk factors and work-related musculoskeletal disorders in construction workers based on literature in the last 20 years: A systematic review. Int. J. Ind. Ergon..

[15] Nicoletti S., Carino M., Di Leone G., Trani G., Carella F., Rubino G., Leone E., Popolizio R., Colafiglio S., Ambrosi L. (2008). Prevalence of upper limb work-related musculoskeletal disorders (UL-WMSDs) in workers of the upholstered furniture industry. La Med. Del Lav..

[16] Nicoletti S., Carino M., Di Leone G., Trani G., Colombini D., Occhipinti E. (2008). Risk assessment of work-related upper limb musculoskeletal disorders in thirty factories in the upholstered furniture industry. La Med. Del Lav..

[17] Esposito C., Najmaldin A., Schier F., Yamataka A., Ferro M., Riccipetitoni G., Czauderna P., Ponsky T., Till H., Escolino M. (2014). Work-related upper limb musculoskeletal disorders in pediatric minimally invasive surgery: A multicentric survey comparing laparoscopic and sils ergonomy. Pediatr. Surg. Int..

[18] Berguer R., Rab G.T., Abu-Ghaida H., Alarcon A., Chung J. (1997). A comparison of surgeons’ posture during laparoscopic and open surgical procedures. Surg. Endosc..

[19] Zahiri H.R., Addo A., Park A.E. (2019). Musculoskeletal Disorders in Minimally Invasive Surgery. Adv. Surg..

[20] Davila V., Meltzer A., Fortune E., Morrow M., Lowndes B., Linden A., Hallbeck M., Money S. (2021). Intraprocedural ergonomics of vascular surgeons. J. Vasc Surg..

[21] Ayad T., Péloquin L., Prince F. (2005). Ergonomics in endoscopic sinus surgery: Systematic review of the literature. J. Otolaryngol..

[22] Savoie S., Tanguay S., Centomo H., Beauchamp G., Anidjar M., Prince F. (2007). Postural control during laparoscopic surgical tasks. Am. J. Surg..

[23] Esposito C., Ghoneimi A.E., Yamataka A., Rothenberg S., Bailez M., Ferro M., Gamba P., Castagnetti M., Mattioli G., Delagausie P. (2013). Work-related upper limb musculoskeletal disorders in paediatric laparoscopic surgery. A multicenter survey. J. Pediatr. Surg..

[24] Nakayashiki A., Kawaguchi T., Nakagawa A., Mochizuki F., Furukawa H., Nagai A., Suematsu T., Tominaga T. (2019). Reducing Surgeon’s Physical Stress in Minimally Invasive Neurosurgery. J. Neurol. Surg. A Cent. Eur. Neurosurg..

[25] Asadi H., Monfared S., Athanasiadis D.I., Stefanidis D., Yu D. (2021). Continuous, integrated sensors for predicting fatigue during non-repetitive work: Demonstration of technique in the operating room. Ergonomics.

[26] World Medical Association (2013). World Medical Association Declaration of Helsinki: Ethical Principles for Medical Research Involving Human Subjects. JAMA.

[27] Sambandam S.N., Khanna V., Gul A., Mounasamy V. (2015). Rotator cuff tears: An evidence based approach. World J. Orthop..

[28] Onks C., Silvis M., Loeffert J., Tucker J., Gallo R.A. (2020). Conservative care or surgery for rotator cuff tears?. J. Fam. Pract..

[29] Longo U.G., Risi Ambrogioni L., Candela V., Berton A., Carnevale A., Schena E., Denaro V. (2021). Conservative versus surgical management for patients with rotator cuff tears: A systematic review and META-analysis. BMC Musculoskelet. Disord..

[30] Stegeman D.F., Merletti R., Hermens H.J. (2004). EMG Modeling and Simulation. Electromyography: Physiology, Engineering, and Noninvasive Applications.

[31] Whittaker R.L., La Delfa N.J., Dickerson C.R. (2019). Algorithmically detectable directional changes in upper extremity motion indicate substantial myoelectric shoulder muscle fatigue during a repetitive manual task. Ergonomics.

[32] Norasi H., Tetteh E., Money S., Davila V., Meltzer A., Morrow M., Fortune E., Mendes B., Hallbeck M. (2021). Intraoperative posture and workload assessment in vascular surgery. Appl. Ergon..

[33] Carbonaro N., Mascherini G., Bartolini I., Ringressi M., Taddei A., Tognetti A., Vanello N. (2021). A wearable sensor-based platform for surgeon posture monitoring: A tool to prevent musculoskeletal disorders. Int. J. Environ. Res. Public Health.

[34] Nowakowski M., Trybek P., Rubinkiewicz M., Cegielny T., Romaniszyn M., Pędziwiatr M., Machura Ł. (2018). Upper extremity surface electromyography signal changes after laparoscopic training. Wideochir Inne Tech. Maloinwazyjne.

[35] Fan X., Forsman M., Yang L., Lind C., Kjellman M. (2022). Surgeons’ physical workload in open surgery versus robot-assisted surgery and nonsurgical tasks. Surg. Endosc..

[36] Judkins T., Oleynikov D., Narazaki K., Stergiou N. (2006). Robotic surgery and training: Electromyographic correlates of robotic laparoscopic training. Surg. Endosc..

[37] Gutierrez-Diez M.C., Benito-Gonzalez M.A., Sancibrian R., Gandarillas-Gonzalez M.A., Redondo-Figuero C., Manuel-Palazuelos J.C. (2018). A study of the prevalence of musculoskeletal disorders in surgeons performing minimally invasive surgery. Int. J. Occup. Saf. Ergon..

[38] Sousa A.S., Tavares J.M.R., Hiroki T. (2012). Surface electromyographic amplitude normalization methods: A review. Electromyography: New Developments, Procedures and Applications.

[39] van Hedel H., Tomatis L., Müller R. (2006). Modulation of leg muscle activity and gait kinematics by walking speed and bodyweight unloading. Gait Posture.

[40] Sözen H., Akyıldız C. (2018). The Effects of Aerobic and Anaerobic Training on Aerobic and Anaerobic Capacity. J. Int. Anatolia Sport Sci..

[41] Kahn J.F., Monod H. (1989). Fatigue induced by static work. Ergonomics.

[42] Murthy G., Hargens A.R., Lehman S., Rempel D.M. (2001). Ischemia causes muscle fatigue. J. Orthop. Res..

[43] Scarlet S., Dreesen E. (2020). Should anesthesiologists and surgeons take breaks during cases?. AMA J. Ethics.

[44] Engelmann C., Schneider M., Grote G., Kirschbaum C., Dingemann J., Osthaus A., Ure B. (2012). Work breaks during minimally invasive surgery in children: Patient benefits and surgeon’s perceptions. Eur. J. Pediatr. Surg..

[45] Janhofer D., Lakhiani C., Song D. (2019). Addressing surgeon fatigue: Current understanding and strategies for mitigation. Plast. Reconstr. Surg..

[46] Park A., Zahiri H., Hallbeck M., Augenstein V., Sutton E., Yu D., Lowndes B., Bingener J. (2017). Intraoperative “Micro Breaks” with targeted stretching enhance surgeon physical function and mental focus: A multicenter cohort study. Ann. Surg..

